# Integrated analysis of serum lipid profile for predicting clinical outcomes of patients with malignant biliary tumor

**DOI:** 10.1186/s12885-020-07496-8

**Published:** 2020-10-09

**Authors:** Lejia Sun, Xin Ji, Dongyue Wang, Ai Guan, Yao Xiao, Haifeng Xu, Shunda Du, Yiyao Xu, Haitao Zhao, Xin Lu, Xinting Sang, Shouxian Zhong, Huayu Yang, Yilei Mao

**Affiliations:** 1 Department of Liver Surgery, Peking Union Medical College (PUMC) Hospital, PUMC & Chinese Academy of Medical Sciences, Beijing, 100730 China; 2 Peking Union Medical College (PUMC), PUMC & Chinese Academy of Medical Sciences, Beijing, 100730 China

**Keywords:** Serum lipid, Prognosis, Biomarker, Nomogram, Malignant biliary tumor

## Abstract

**Background:**

Serum lipids were reported to be the prognostic factors of various cancers, but their prognostic value in malignant biliary tumor (MBT) patients remains unclear. Thus we aim to assess and compare prognosis values of different serum lipids, and construct a novel prognostic nomogram based on serum lipids.

**Methods:**

Patients with a confirmed diagnosis of MBT at our institute from 2003 to 2017 were retrospectively reviewed. Prognosis-related factors were identified via univariate and multivariate Cox regression analyses. Then the novel prognostic nomogram and a 3-tier staging system were constructed based on these factors and further compared to the TNM staging system.

**Results:**

A total of 368 patients were included in this study. Seven optimal survival-related factors—TC/HDL >  10.08, apolipoprotein B >  0.9 g/L, lipoprotein> 72 mg/L, lymph node metastasis, radical cure, CA199 > 37 U/mL, and tumor differentiation —were included to construct the prognostic nomogram. The C-indexes in training and validation sets were 0.738 and 0.721, respectively. Besides, ROC curves, calibration plots, and decision curve analysis all suggested favorable discrimination and predictive ability. The nomogram also performed better predictive ability than the TNM system and nomogram without lipid parameters. And the staging system based on nomogram also presented better discriminative ability than TNM system (*P* < 0.001).

**Conclusions:**

The promising prognostic nomogram based on lipid parameters provided an intuitive method for performing survival prediction and facilitating individualized treatment and was a great complement to the TNM staging system in predicting overall survival.

## Background

Malignant biliary tumor (MBT) is amongst the most aggressive gastrointestinal tract tumor and accounts for approximately 4% of all malignancy in the gastrointestinal tract [1]. MBT received considerable attention for its high mortality, poor prognosis and great repercussion on quality of life, with a 5-year overall survival (OS) rate of 25–50% for gallbladder cancer (GBC), 20–40% for intrahepatic cholangiocarcinoma (ICC) and 15–50% for extrahepatic cholangiocarcinoma (ECC) [2, 3]. However, due to the silent clinical manifestations, low specificity of most diagnostic modalities, high invasiveness, early hematogenous and lymphatic metastasis, almost 30–70% of patients are not capable of curative treatment [3–5]. Thus, new prognostic factors with good predictive ability and the prognostic predictive model is needed for early risk stratification of patients’ prognosis as well as identifying potential therapeutic targets, which further facilitate early clinical intervene and individualized treatment.

Tumor staging is essential for MBT because the choice of therapeutic options largely depends on the stage. And inaccurate staging could cause patients to suffer extra injuries or lose their possibility to cure as the surgery is the only potentially curative treatment. Currently, prognostic prediction of MBT mostly based on the AJCC TNM staging system [6], which mainly based on surgical pathology features including tumor size, lymph node metastasis and distant metastasis, regardless of clinical and laboratory parameters. Previous studies also proposed several staging systems including clinical parameters such as hemoglobin, alkaline phosphatase and C reactive protein, but showed a suboptimal correlation with survival or lack of validation, such as the GBC staging system created by Yadav S, et al. [7]. Therefore, new prognostic factors with better predictive capability for BMT patients are needed.

Abnormal lipid metabolism has been reported to play a vital role in tumorigenesis, progression and metastasis through different pathways. For example, abnormal triglyceride metabolism was reported to regulate cell proliferation through AMPK- mTOR pathway, and cholesterol could promote tumor progression and chemotherapy resistance through altering cytoskeleton, angiogenesis and cell apoptosis [8, 9]. Moreover, several lipid parameters were reported to be the efficient prognostic factors of various cancer, such as breast cancer, gastric cancer, hepatocellular carcinoma, etc. [10–12]. However, no study has evaluated the clinical significance and the potential prognostic role of serum lipid profile in MBT patients.

Thus, in the present study, we investigated the prognostic value of serum lipids in BMT, and developed a novel, promising prognostic nomogram based on serum lipid parameters and clinicopathological parameters. Furthermore, we created a new staging system based on the nomogram and validated its efficacy in predicting prognosis in BMT patients treated in a tertiary referral center.

## Methods

### Patients

All the patients who had documented cases of MBT in the Peking Union Medical College Hospital (PUMCH) database between January 2003 and December 2017 were retrospectively reviewed. Patients were included if they 1) received surgery and histopathologically confirmed MBT; 2) post-surgery follow-up more than 3 months; 3) relatively complete preoperative examination. And patients were excluded if they 1) lack of serum lipids examination; 2) with an AJCC TNM stage of 4; 3) with a history of other malignant tumors.

### Data collection and definition

Baseline information including demographic data, history of alcohol and fatty liver, jaundice, tumor condition, therapeutic options, laboratory data, and overall survival (OS) were obtained from medical records. Tumor condition included tumor size, extent, lymph node metastasis, and histopathological data. Therapeutic options included radical cure and palliative resection, of which radical cure was defined as fully clearing of gross lesion, lymphadenectomy, and histologically negative (R0) surgical margin. Blood samples were obtained from all patients after over 12 h overnight fasting, and all samples were immediately transferred to the laboratory for batch analysis. Laboratory data including hepatic parameters (including albumin, alanine aminotransferase (ALT), aspartate aminotransferase (AST), gamma-glutamyl transpeptidase (GGT), alkaline phosphatase (ALP), lactate dehydrogenase (LDH), total bilirubin (TBIL)), serum lipid assessment (including total cholesterol (TC), triglycerides (TG), high-density lipoprotein (HDL), low-density lipoprotein (LDL), ratio of TC to HDL (TC/HDL), ratio of TG to HDL (TG/HDL), apolipoprotein A-1 (ApoA1), apolipoprotein B (ApoB) and lipoprotein) and CA199 level were obtained at the time of diagnosis of MBT.

### Statistical analysis

Descriptive data are expressed as mean ± SD, median (interquartile range, IQR) or frequencies (%), respectively. Normally and non-normally distributed quantitative variables were compared using two-sample *t*-test and Mann-Whitney U test, respectively. Categorical variables were compared using Fisher’s exact test or Chi-Square test according to sample size. The cutoff value for body mass index (BMI), hepatic parameters and CA199 were defined according to the reference range in the clinical application. And for serum lipid parameters, the cut-off value was calculated through R package ‘maxstat’, which could divide cohorts into 2 groups with maximum differences of the log-rank test results for overall survival [13].

To identify survival-associated parameters, univariate Cox regression was first performed using R package ‘survival’ in the whole cohort (*n* = 368) [14]. And ‘timeROC’ package was used to further evaluate the prognostic ability of various serum lipid parameters. Then we performed multivariate COX analysis in the modified cohort (*n* = 252) which excluded patients with incomplete data of serum lipid, hepatic parameters or CA199 to identified independent prognostic factors. Subsequently, we performed the proportional-hazards assumption test based on Schoenfeld residuals to exclude time-dependent factors. Then we randomly separated the 252 patients into training set (*n* = 152) and validation set (*n* = 100) with the ratio of 3:2. And the independent prognostic factors were included to construct a novel prognostic nomogram using the ‘rms’ R package based on the training set [15]. The discriminative ability of the nomogram was evaluated by the Harrell’s concordance index (C-index), time-dependent receiving operative characteristics (ROC) curve. Moreover, calibration plots, integrated discrimination improvement (IDI), decision curve analysis (DCA) were performed to graphically evaluate the consistency and efficacy of the nomogram [16]. Furthermore, the prognostic nomogram was evaluated in the validation set using the same methods.

We further calculated the risk score of each patients based on the nomogram, then constructed a new staging system. To compare the prognostic predictive ability between our nomogram-based staging system and TNM staging system, Kaplan-Meier (K-M) OS curves were constructed to estimate the prognoses of patients, and the survival differences among subgroups were assessed by a two-sided log-rank test. C-indexes of various staging systems were compared using the likelihood-ratio test. All analyses were performed with R version 3.6.3 (The R Foundation for Statistical Computing, Vienna, Austria) and Statistical Package for the Social Sciences (SPSS) software version 26 (SPSS Inc., Chicago, IL, USA), and a two-tailed *P*-value of < 0.05 was considered statistically significant. Hazard ratios (HRs) and 95% confidence intervals (CIs) were reported if necessary.

## Results

### Patient characteristics

A total of 368 patients met the selection criteria were included in this study, with 73 GBC, 74 ICC and 221 ECC. The median age at diagnosis was 62 (IQR, 56–69) years, and 168 patients (45.7%) were female. Besides, the proportion of the patients at TNM stage I, II, III were 36.1, 32.6 and 31.3%, respectively. And 248 patients (67.4%) had R0 surgical margin, with 232 patients (63.0%) received a radical cure. The median overall survival was 20 (IQR, 10.7–36.3) months. Table 1 showed the detailed demographic and clinicopathological features of the cohort. And the median overall survival of the training set (*n* = 152) and validation set (*n* = 100) were 18.9 (10.2–34.2) months, 24.9 (11.1–39.4) months, respectively (Additional file 1: Table S1). Besides, in the training set, 35.5% was at TNM stage I, 30.3% at stage II, 34.2% at stage III, and 63.2% of the patients received radical cure. Consistently, in the validation set, 40.0% was at TNM stage I, 27.0% at stage II, 33.0% at stage III, and 61.0% of the patients received radical cure.

**Table 1:** Baseline characteristics

Characteristics	n (%)
Total, N	368(100)
Tumor type	
GBC	73 (19.8)
ICC	74 (20.1)
ECC	221 (60.0)
Age (years)	
≤ 60	155 (42.1)
> 60	213 (57.9)
Sex	
Female	168 (45.7)
Male	200 (54.3)
BMI at diagnosis, kg/m^2^	
< 18.5	19 (5.2)
18.5–22.9	128 (34.8)
≥ 23	177 (48.1)
NA	44 (12.0)
Alcohol	
Yes	90 (24.5)
No	278 (75.5)
Fatty liver	
Yes	27 (7.3)
No	341 (92.7)
Jaundice	
Yes	219 (59.5)
No	149 (40.5)
Tumor size, cm	
D ≤ 2	180 (50.6)
2 < D ≤ 3	85 (23.1)
3 < D ≤ 4	35 (9.5)
D > 4	56 (15.2)
NA	12 (3.3)
Lymph node metastasis	
Yes	122 (33.2)
No	246 (66.8)
Extrahepatic involvement	
Yes	252 (68.5)
No	116 (31.5)
Intrahepatic involvement	
Yes	111 (30.2)
No	257 (69.8)
Gallbladder involvement	
Yes	91 (24.7)
No	277 (75.3)
AJCC 8th TNM stage	
I	133 (36.1)
II	120 (32.6)
III	115 (31.3)
Tumor differentiation	
Undifferentiation	2 (0.5)
Low	36 (9.8)
Low-moderate	98 (26.6)
Moderate	102 (27.7)
Moderate-high	28 (7.6)
High	102 (27.7)
Radical cure	
Yes	232 (63.0)
No	136 (37.0)
R0	
Yes	248 (67.4)
No	120 (32.6)
TC level, mmol/L	
≤ 7.28	301 (81.8)
> 7.28	67 (18.2)
TG level, mmol/L	
≤ 3.14	301 (81.8)
> 3.14	67 (18.2)
HDL level, mmol/L	
≤ 0.95	205 (55.7)
> 0.95	163 (44.3)
LDL level, mmol/L	
≤ 1.96	41 (11.1)
> 1.96	327 (88.9)
TC/HDL	
≤ 10.08	256 (69.6)
> 10.08	112 (30.4)
TG/HDL	
≤ 4.16	258 (70.1)
> 4.16	110 (29.9)
ApoA1 level, g/L	
≤ 1.11	166 (45.1)
> 1.11	150 (40.8)
NA	52 (14.1)
ApoB level, g/L	
≤ 0.90	210 (57.1)
> 0.90	105 (28.5)
NA	53 (14.4)
Lipoprotein level, mg/L	
≤ 72	152 (41.3)
> 72	118 (32.1)
NA	98 (26.6)
Albumin level, g/L	
≤ 35	59 (16.0)
> 35	305 (82.9)
NA	4 (1.1)
ALT level, U/L	
≤ 40	116 (31.5)
> 40	252 (68.5)
AST level, U/L	
≤ 40	130 (35.3)
> 40	233 (63.3)
NA	5 (1.4)
GGT level, U/L	
≤ 45	72 (19.5)
> 45	291 (79.1)
NA	5 (1.4)
ALP level, U/L	
≤ 135	121 (32.9)
> 135	242 (65.7)
NA	5 (1.4)
LDH level, U/L	
≤ 250	300 (81.5)
> 250	61 (16.6)
NA	7 (1.9)
TBIL level, umol/L	
≤ 17.1	113 (30.7)
> 17.1	255 (69.3)
CA199 level, U/mL	
≤ 37	94 (25.5)
> 37	263 (71.5)
NA	11 (3.0)
Overall survival, months	21.9 (11.0–39.0)

*ALP* Alkaline phosphatase, *ALT* Alanine aminotransferase, *ApoA1* ApolipoproteinA-1, *APOB* Apolipoprotein B, *AST* Aspartate aminotransferase, *BMI* Body mass index, *CA199* Carbohydrate antigen 199, *CC* Cholangiocarcinoma, *D* maximum diameter of tumor, *ECC* Extrahepatic cholangiocarcinoma, *GBC* Gallbladder cancer, *GGT* Gamma-glutamyl transpeptidase, *HDL* High-density lipoprotein, *ICC* Intrahepatic cholangiocarcinoma, *LDH* Lactate dehydrogenase, *LDL* low-density lipoprotein, *NA* Not accessible, *R0* Histologically negative surgical margin, *TBIL* Total bilirubin, *TC* Total cholesterol, *TG* Triglycerides

### Association between serum lipids and survival time

**Fig. 1:**
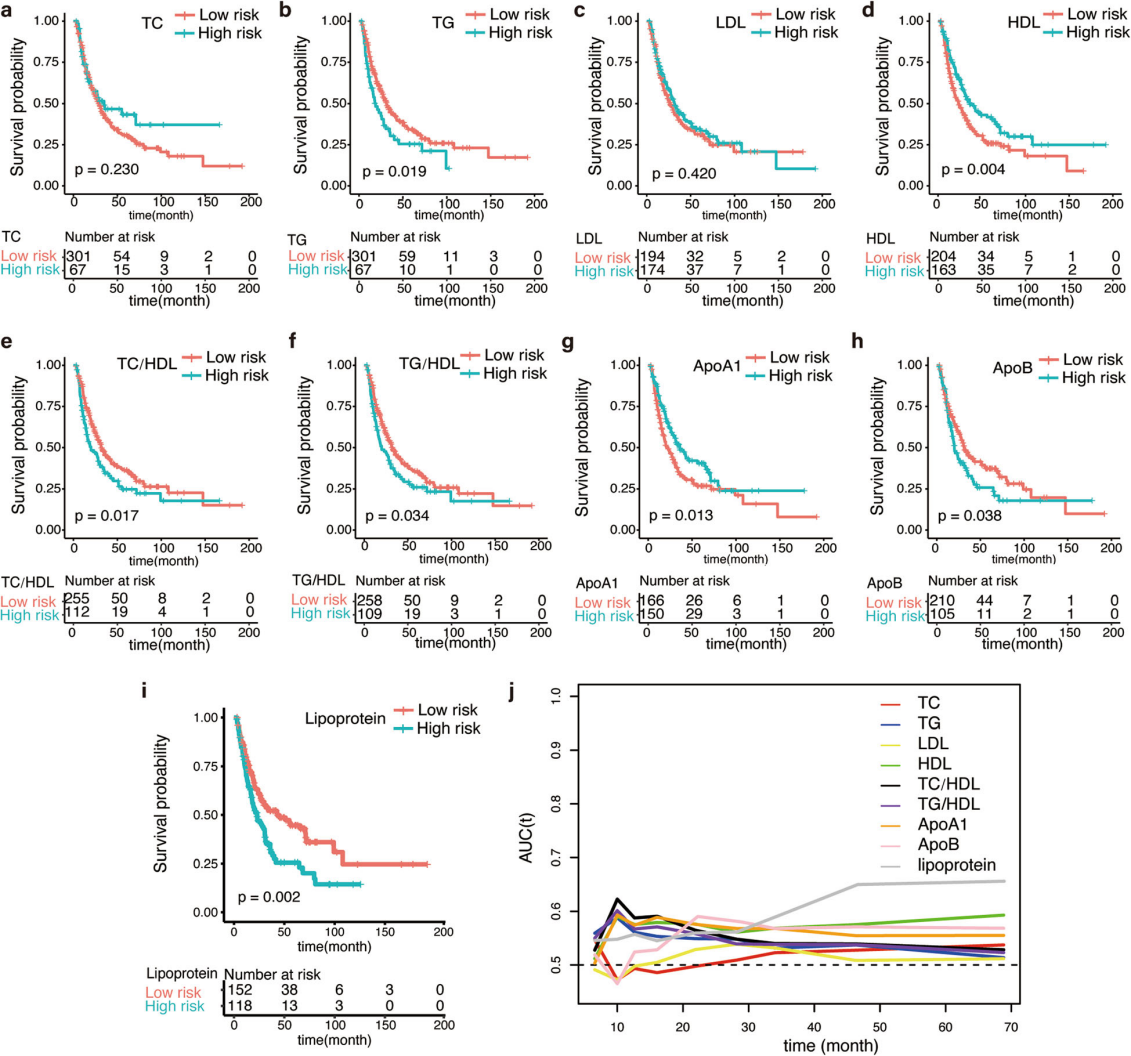
K-M survival curves and time-dependent ROC curves for lipid parameters. K-M survival curves show the overall survival rates in MBT patients according to TC (**a**), TG (**b**), LDL (**c**), HDL (**d**), TC/HDL (**e**), TG/HDL (**f**), ApoA1 (**g**), ApoB (**h**), lipoprotein (**i**) subgroups. Red lines indicate high-risk groups, blue lines indicate low-risk groups. *P*-values are shown in the bottom left corner. Time-dependent numbers at risk are listed at the bottom. And time-dependent ROC curves show the AUC of each lipid parameters (**j**). K-M, Kaplan-Meier; ROC, receiving operative characteristics; MBT, malignant biliary tumor; TC, total cholesterol; TG, triglycerides; HDL, high-density lipoprotein; LDL, low-density lipoprotein; ApoA1, apolipoprotein A-1; ApoB, apolipoprotein B

The cut-off value of TC, TG, HDL, LDL, TC/HDL, TG/HDL, ApoA1, ApoB and lipoprotein were 7.28 mmol/L, 3.14 mmol/L, 0.95 mmol/L, 1.96 mmol/L, 10.08, 4.16, 1.11 g/L, 0.90 g/L, 72 mg/L, respectively. Then we defined the group with serum lipid parameters higher than the cut-off value as high-risk group, and low-risk group if not. High level of TG, TC/HDL, TG/HDL, ApoB and lipoprotein showed significantly poorer overall survival (Fig. 1a-c, e, f, h, i). Besides, HDL and ApoA1 showed significantly better prognosis in the high-risk group when compared with low-risk group (Fig. 1d, g). The results of time-dependent ROC curve for OS showed that TC and LDL performed relatively poor predictive performance, while TC/HDL, ApoB and lipoprotein exhibited favorable discriminative ability (Fig. 1j). Especially, lipoprotein presented an obviously better discriminative ability than other parameters after 30 months, ApoB presented a relatively good discriminative capability during 20–30 months, and TC/HDL showed the optimal predictive ability within 20 months after surgery.

### The prognostic factors of patients with MBT

The univariable analysis demonstrated that BMI, jaundice, lymph node metastasis, intrahepatic involvement, extrahepatic involvement, tumor differentiation, radical cure, R0, TG, HDL, TC/HDL, TG/HDL, ApoA1, ApoB, lipoprotein, albumin, GGT, ALP, TBIL, CA199 were associated with OS (*P* < 0.05) (Table 2). We further performed multivariate COX analysis in the training set to identify independent prognostic factors. And lymph node metastasis (*P* = 5.88 × 10^− 7^), tumor differentiation (*P* = 0.023), radical cure (*P* = 4.87 × 10^− 7^), R0 (*P* = 0.025), TC/HDL (*P* = 0.049), ApoB (*P* = 7.12 × 10^− 7^), lipoprotein (*P* = 1,12 × 10^− 6^), CA199 (*P* = 0.007) were shown to determine the OS (Table 2). We further performed proportional-hazards assumption test based on Schoenfeld residuals and excluded R0 for it was a time-dependent factor (*P* < 0.05) (Additional file 2: Figure S1).

**Table 2:** Univariate and multivariate cox proportional hazards analysis of clinicopathological variables

Characteristics	Univariate COX analysis (*n* = 368)		Multivariate COX analysis (*n* = 252)	
	HR (95% CI)	*P* value	HR (95% CI)	*P* value
Age, years (≤60/> 60)	1.126(0.860–1.473)	0.388	–	–
Sex (Female/Male)	0.963(0.738–1.256)	0.780	–	–
BMI, kg/m^2^ (< 18.5/18.5–23/≥23)	0.734(0.588–0.916)	**0.006**	0.913(0.594–1.405)	0.680
Alcohol (No/Yes)	1.088(0.803–1.474)	0.586	–	–
Fatty liver (No/Yes)	1.211(0.745–1.965)	0.438	–	–
Jaundice (No/Yes)	1.547(1.17–2.045)	**0.002**	1.441(0.709–2.931)	0.313
Cardiovascular diseases (No/Yes)	0.966(0.736–1.267)	0.801	–	–
Tumor size, cm (≤2/2–3/3–4/> 5)	1.028(0.913–1.158)	0.646	–	–
Lymph node metastasis (No/Yes)	2.301(1.752–3.022)	**2.07e-09**	2.776(1.860–4.144)	**5.88e-07**
Extrahepatic involvement (No/Yes)	1.398(1.04–1.878)	**0.026**	1.277(0.614–2.656)	0.513
Intrahepatic involvement (No/Yes)	1.386(1.047–1.835)	**0.022**	1.491(0.944–2.354)	0.087
Gallbladder involvement (No/Yes)	1.170(0.861–1.591)	0.314		
Tumor differentiation (low/low-moderate/moderate/moderate-high/high/undefined)	0.781(0.705–0.865)	**2.01e-06**	0.840(0.723–0.977)	**0.023**
Radical cure (No/Yes)	0.381(0.292–0.498)	**1.35e-12**	0.285(0.174–0.464)	**4.89e-07**
R0 (No/Yes)	0.643 (0.493–0.839)	**0.001**	1.681(1.069–2.643)	**0.025**
TC (≤4.97/> 4.97)	0.800(0.552–1.158)	0.236	–	–
TG (≤0.9/> 0.9)	1.477(1.063–2.051)	**0.020**	1.022(0.456–2.288)	0.959
HDL (≤0.94/> 0.94)	0.676(0.516–0.886)	**0.004**	1.026(0.611–1.722)	0.924
LDL (≤2.18/> 2.18)	0.790(0.534–1.168)	0.238	–	–
TC/HDL (≤11.4/> 11.4)	1.404(1.061–1.858)	**0.018**	2.330(1.005–5.404)	**0.049**
TG/HDL (≤4.16/> 4.16)	1.355(1.022–1.797)	**0.034**	0.550(0.230–1.314)	0.178
ApoA1 (≤0.72/> 0.72)	0.694(0.520–0.927)	**0.013**	0.967(0.571–1.636)	0.900
ApoB (≤0.9/> 0.9)	1.368(1.051–1.842)	**0.039**	2.931(1.916–4.483)	**7.12e-07**
Lipoprotein (≤89/> 89)	1.630(1.188–2.235)	**0.002**	2.635(1.784–3.891)	**1.12e-06**
Albumin (≤35/> 35)	0.965(0.938–0.992)	**0.001**	1.157(0.686–1.953)	0.585
ALT (≤40/> 40)	1.339(0.995–1.802)	0.054	–	–
AST (≤40/> 40)	1.252(0.941–1.665)	0.123	–	–
GGT (≤45/> 45)	1.968(1.345–2.881)	**4.96e-04**	0.822(0.444–1.522)	0.534
ALP (≤135/> 135)	1.634(1.211–2.205)	**0.001**	0.734(0.386–1.397)	0.346
LDH (≤250/> 250)	1.401(0.996–1.971)	0.052	–	–
TBIL (≤17.1/> 17.1)	1.619(1.183–2.216)	**0.002**	1.311(0.720–2.385)	0.376
CA199 (≤37/> 37)	2.408(1.675–3.463)	**2.12e-06**	2.043(1.210–3.447)	**0.007**

All statistical tests were two-sided. Bold type means *P* < 0.05

*ALP* Alkaline phosphatase, *ALT* Alanine aminotransferase, *ApoA1* ApolipoproteinA-1, *APOB* Apolipoprotein B, *AST* Aspartate aminotransferase, *BMI* Body mass index, *CA199* Carbohydrate antigen 199, *GGT* Gamma-glutamyl transpeptidase, *HDL* High-density lipoprotein, *ICC* Intrahepatic cholangiocarcinoma, *LDH* Lactate dehydrogenase, *LDL* Low-density lipoprotein, *R0* Histologically negative surgical margin, *TBIL* Total bilirubin, *TC* Total cholesterol, *TG* Triglycerides

### Association among lipid prognostic factors, clinicopathological features and surgical outcome

To better understand the roles of serum lipid in the prognosis of MBT patients, we further analyzed the relationships among the 3 lipid prognostic factors and the clinicopathological characteristics as well as the surgical outcomes of MBT patients (Table 3). Specially, TC/HDL, ApoB and lipoprotein all related to tumor type and jaundice, and not associated with alcohol, fatty liver, cardiovascular diseases, lymph node metastasis, radical cure and blood loss during surgery. Patients with high level of TC/HDL tend to have lower BMI, tumor larger than 4 cm, and more extrahepatic involvement but less intrahepatic involvement, gallbladder involvement and less proportion of high differentiation. What’s more, high-level TC/HDL patients might have longer postoperative hospital days, with a higher probability of postoperative bleeding. For patients with a high level of ApoB, they might more likely to be overweight and have hepatic and gallbladder involvement. While high lipoprotein level related to overweight, extrahepatic involvement, more postoperative bleeding and infection.

**Table 3:** Association among serum lipids and clinicopathological parameters and surgical outcomes

	TC/HDL			ApoB, g/L			Lipoprotein, g/L		
	≤ 10.08 (***n*** = 187)	> 10.08 (***n*** = 65)	p	≤ 0.9 (***n*** = 87)	> 0.9 (***n*** = 165)	p	≤ 72 (***n*** = 141)	> 72 (***n*** = 111)	p
Clinicopathological characteristics									
Tumor type			< 0.001			< 0.001			0.004
GBC	64(34.2)	2(3.1)	< 0.001	30(34.5)	36(21.8)	0.030	32(22.7)	34(30.6)	0.155
ICC	42(22.5)	5(7.7)	0.008	25(28.7)	22(13.3)	0.003	20(14.2)	27(24.3)	0.040
ECC	81(43.3)	58(89.2)	< 0.001	32(36.8)	107(64.8)	< 0.001	89(63.1)	50(45.0)	0.004
BMI ≥ 23 kg/m^2^	122(65.2)	3(4.6)	< 0.001	57(65.5)	68(41.2)	< 0.001	59(41.8)	66(59.5)	0.005
Alcohol	40(21.4)	21(32.3)	0.077	17(19.5)	44(26.7)	0.209	32(22.7)	29(26.1)	0.528
Fatty liver	8(4.3)	5(7.7)	0.455	2(2.3)	11(6.7)	0.234	9(6.4)	4(3.6)	0.322
Jaundice	69(36.9)	64(98.5)	< 0.001	32(36.8)	101(61.2)	< 0.001	87(61.7)	46(41.4)	0.001
Cardiovascular disease	77(41.2)	25(38.5)	0.701	33(38.0)	69(41.8)	0.550	57(40.4)	45(40.5)	0.985
Tumor size, cm			0.029			0.882			0.637
d ≤ 2	78(41.7)	34(52.3)	0.139	35(40.2)	77(46.7)	0.328	68(48.2)	44(39.6)	0.173
2 < d ≤ 3	40(21.4)	19(29.2)	0.198	21(24.1)	38(23.0)	0.843	32(22.7)	27(24.3)	0.762
3 < d ≤ 4	21(11.2)	6(9.2)	0.653	10(11.5)	17(10.3)	0.771	15(10.6)	12(10.8)	0.965
d > 4	40(21.4)	4(6.2)	0.005	16(18.4)	28(17.0)	0.778	22(15.6)	22(19.8)	0.381
Lymph node metastasis	58(31.0)	23(35.4)	0.516	25(28.7)	56(33.9)	0.400	42(29.8)	39(35.1)	0.367
Extrahepatic involvement	96(51.3)	62(95.4)	< 0.001	42(48.3)	116(70.3)	0.001	100(70.9)	58(52.3)	0.002
Intrahepatic involvement	67(35.8)	14(21.5)	0.034	35(40.2)	46(27.9)	0.046	40(28.4)	41(36.9)	0.148
Gallbladder involvement	69(36.9)	9(13.8)	0.001	36(41.4)	42(25.5)	0.009	39(27.7)	39(35.1)	0.203
TNM			< 0.001			0.018			0.166
I	69(36.9)	25(38.5)	0.822	32(36.8)	62(37.6)	0.901	52(36.9)	42(37.8)	0.876
II	43(23.0)	30(46.2)	< 0.001	17(19.5)	56(33.9)	0.017	47(33.3)	26(23.4)	0.085
III	75(40.1)	10(15.4)	< 0.001	38(43.7)	47(28.5)	0.015	42(29.8)	43(38.7)	0.136
High differentiation	62(33.2)	13(20.0)	0.046	25(28.7)	50(30.3)	0.796	39(27.7)	36(32.4)	0.411
Radical cure	119(63.6)	38(58.5)	0.458	59(67.8)	98(59.4)	0.190	90(63.8)	67(60.4)	0.573
R0	120(64.2)	39(60.0)	0.548	60(70.0)	99(60.0)	0.161	91(64.5)	68(61.3)	0.592
CA199 > 37 U/mL	113(60.4)	60(92.3)	< 0.001	58(66.7)	115(69.7)	0.622	107(75.9)	66(59.5)	< 0.001
Surgical outcome									
Estimated blood loss, mL	300(100–500)	300(200–575)	0.195	300(100–500)	300(200–500)	0.137	400(175–500)	250(150–450)	0.117
Postoperative hospital days	18.0(14.0–26.0)	23.0(20.0–30.5)	< 0.001	18.0(15.0–26.0)	20.5(16.0–28.0)	0.385	20.5(16.0–28.0)	18.0(15.0–27.0)	0.079
Postoperative complications	57(30.5)	24(36.9)	0.338	29(33.3)	52(31.5)	0.622	52(36.9)	29(26.1)	0.070
Bleeding	3(1.6)	6(9.2)	0.014	1(1.1)	8(4.8)	0.251	9(6.4)	0(0.0)	0.018
Infection	29(15.5)	17(26.2)	0.056	13(14.9)	33(20.0)	0.323	32(22.7)	14(12.6)	0.040
Liver dysfunction	0(0.0)	0(0.0)	–	0(0.0)	0(0.0)	–	0(0.0)	0(0.0)	–
Bile leakage	3(1.6)	0(0.0)	0.571	0(0.0)	3(1.8)	0.553	0(0.0)	3(2.7)	0.084
Ascites	17(9.1)	6(9.2)	0.973	10(11.5)	13(7.9)	0.343	14(9.9)	9(8.1)	0.618
Others	24(12.8)	10(15.4)	0.604	14(16.1)	20(12.1)	0.380	20(14.2)	14(12.6)	0.717

Bold type means *P* < 0.05

*ApoB* Apolipoprotein B, *BMI* Body mass index, *CA199* Carbohydrate antigen 199, *CC* Cholangiocarcinoma, *ECC* Extrahepatic cholangiocarcinoma, *GBC* Gallbladder cancer, *ICC* Intrahepatic cholangiocarcinoma, *TC* Total cholesterol

Estimated blood loss and postoperative days were expressed as median (interquartile range, IQR)

### Construction and validation of a novel nomogram for MBT patients

**Fig. 2:**
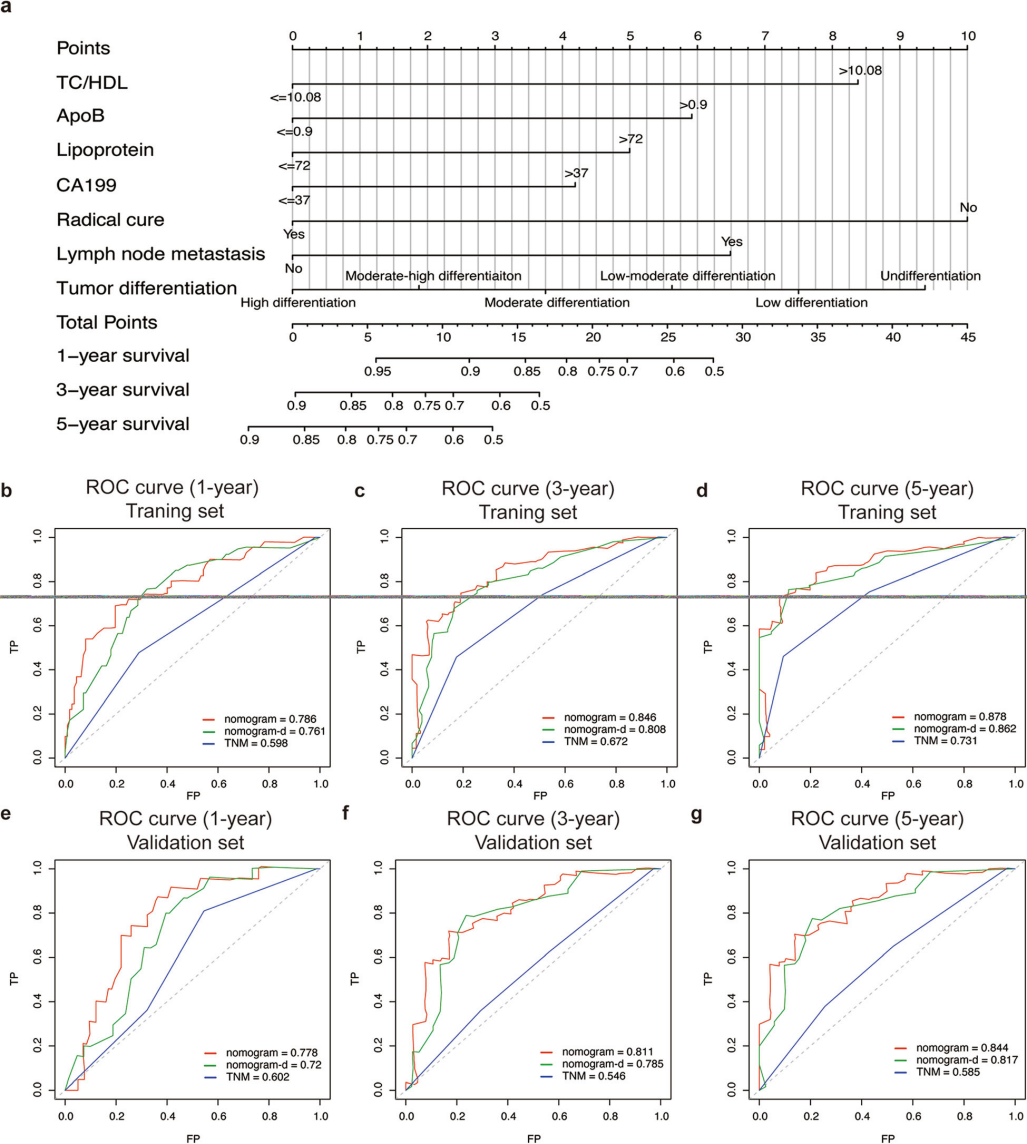
Nomogram for predicting the 1-, 3-, and 5-year survival probability of MBT patients. Prognostic nomogram (**a**) for predicting the survival of MBT patients based on the training set. Comparing ROC curves of the nomogram, nomogram-d and TNM system for 1-, 3-, and 5-year survival in the training set (**b**-**c**) and validation set (**d**-**f**). Nomogram-d deleted serum lipid parameters, which only based on lymph node metastasis, tumor differentiation, radical cure and CA199

**Fig. 3:**
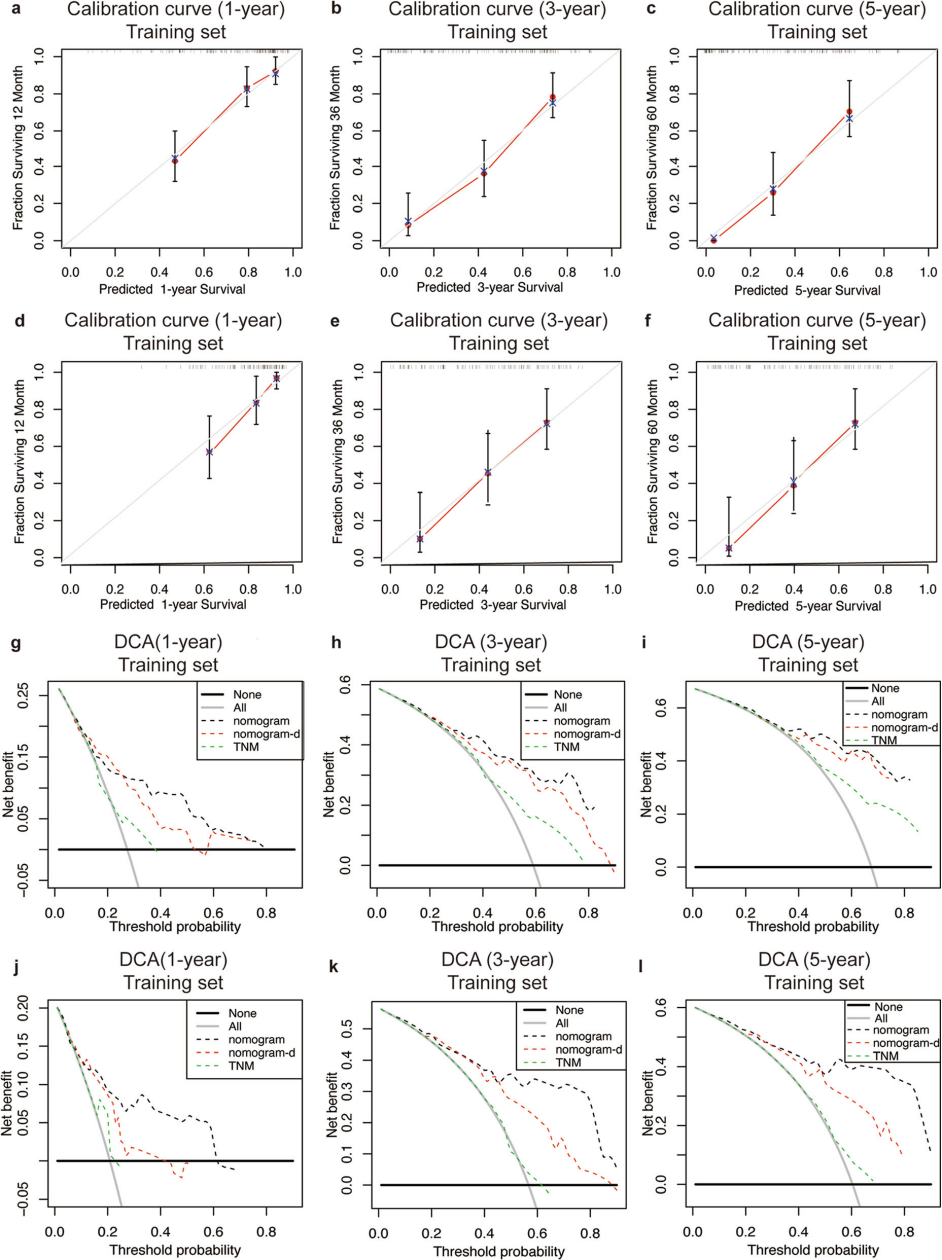
Calibration curves and decision curve analysis of the nomogram. Calibration curves for predicting survival at 1, 3, and 5 years in the training cohort (**a**-**c**) and validation cohort (**d**-**f**). The X-axis indicates the nomogram-predicted probability, and the y-axis indicates actual survival. Time-dependent decision curve analysis for the clinical benefit of the nomograms and the corresponding scope of application in the training set (**g**-**i**) and validation set (**j**-**l**). The grey solid line represents the assumption that all patients survive in first, third and fifth year. The black solid line represents the assumption that no patients survive in the first, third, and fifth year. Nomogram-d was constructed without serum lipid parameters, which only based on lymph node metastasis, tumor differentiation, radical cure and CA199

The prognostic nomogram was then constructed by using the 7 independent prognostic factors previously identified to facilitate clinical prognostic prediction for MBT patients. Figure 2a shows the nomogram predicting the survival probability at 1, 3, and 5 years based on the training set. The C-index of the nomogram for predicting OS was 0.738 (95%CI 0.681–0.795). The AUC values of the 1-, 3-, and 5-year survival predictions using the nomogram were 0.786, 0.846, and 0.878, respectively, indicating a favorable predictive ability (Fig. 2b-d). Besides, when compared to TNM and nomogram-d (nomogram deleted serum lipid parameters, which constructed only based on lymph node metastasis, tumor differentiation, radical cure and CA199), the nomogram presented a higher value of AUC. Consistently, IDI analysis showed adding lipid parameters could significantly improve the discriminative ability of the nomogram (IDI = 0.055, *P* = 0.002). Moreover, the calibration plots (Fig. 3a-c) showed consistency between the nomogram prediction and actual observation of 1-, 3- and 5-year OS in the training set. And DCA (Fig. 3g-i) indicated favorable efficacy of the novel nomogram when compared to the TNM staging system.

In the validation set, the C-index of the nomogram was 0.721 (95%CI 0.660–0.782), AUC values of the 1-, 3-, and 5-year survival predictions using the nomogram were also better than TNM and nomogram-d (Fig. 2e-g), and adding lipid parameters also significantly improved the integrated discriminative ability of the nomogram (IDI = 0.126, *P* < 0.001). Also, both the calibration plots and DCA demonstrated favorable performance for predicting 1-, 3- and 5-year OS in the validation set (Fig. 3d-f and j-i).

### Comparison between the new staging system and AJCC TNM staging system

**Fig. 4:**
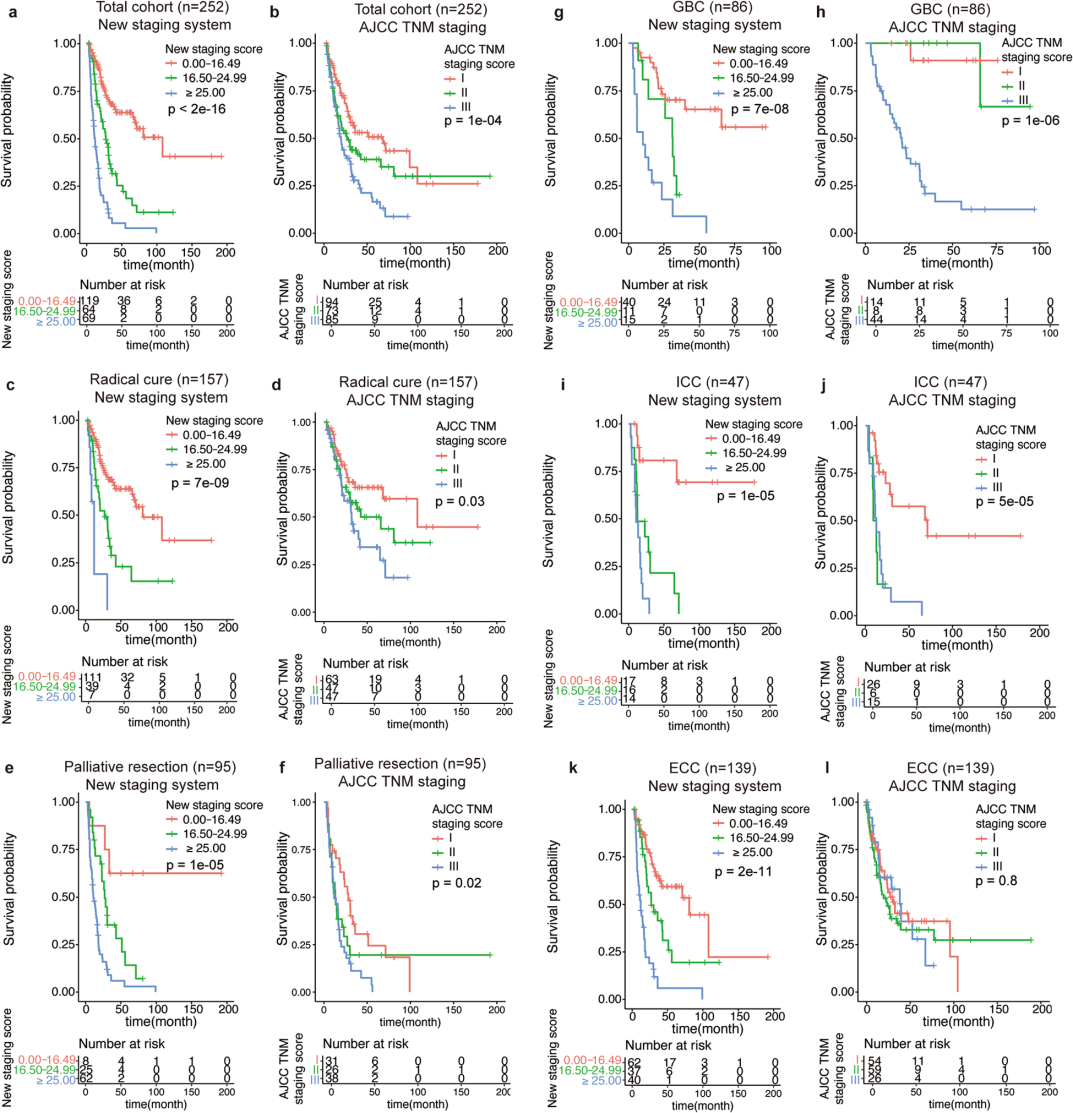
Comparing discriminative ability of two staging system through K-M curves. Overall survival of patients with MBT classified by the new clinical staging system (**a**) and AJCC TNM staging system (**b**) in total cohort; and the 2 systems in radical cure cohort (**c**, **d**), palliative cohort (**e**, **f**), GBC cohort (**g**, **h**), ICC cohort (**i**, **j**), and ECC cohort (**k**, **l**). K-M, Kaplan-Meier; MBT, malignant biliary tumor; GBC, gall bladder cancer; ICC, intrahepatic cholangiocarcinoma; ECC, extrahepatic cholangiocarcinoma

According to the novel nomogram, each patient was assigned a risk score which could indicate individual prognosis. Then we classified the whole cohort into low-risk (*n* = 119), medium-risk (*n* = 64), and high-risk (*n* = 69) groups based on score, with cut-off value of ‘0.00 - 16.49’, ‘16.50 - 24.99’, ‘≥ 25.00’, respectively (Fig. 4a). And KM curves suggested significant differences of prognosis among newly defined 3 groups (*P* < 2.0 × 10^− 16^). While the TNM staging system could not totally separate the prognosis of different TNM stage patients according to the KM OS curves (Fig. 4b). The C-index for the novel staging system was 0.710 (95% CI, 0.670–0.750) compared with 0.588 (95% CI, 0.541–0.634) for the TNM staging system, indicating a better performance in predicting survival (*P* < 0.001).

To further evaluate the efficacy of novel staging system in clinical application, we performed subgroup analysis based on therapeutic options and tumor types. For patients received radical cure, the new staging system performed better discriminative and predictive ability than the AJCC TNM staging system (Fig. 4c-d), with the C-index of 0.637 and 0.547, respectively (*P* = 0.021). For patients received palliative resection, the new staging system also showed better performance than the TNM staging system. And the C-index of the new system was 0.643 compared to 0.582 for TNM system (*P* < 0.001) (Fig. 4e-f).

For GBC patients, the novel staging system showed better ability in distinguishing patients at various stages with log-rank *p*-value of survival differences was 7 × 10^− 8^ (Fig. 4g). While the TNM staging system could hardly distinguish stage I to stage II according to the KM OS curves (Fig. 4h). The C-index of the novel staging system and TNM system were 0.711 and 0.713 (*P* = 0.258). Also, the new staging system exhibited a better performance in distinguishing ICC patients in different stages when compared with the AJCC TNM staging system (Fig. 4i-j). The C-index for the new staging system and TNM staging system were 0.721 and 0.684 (*P* = 0.203). As for ECC patients, the new staging system showed a better discriminative and predictive ability than the TNM system, with the C-index of 0.696 and 0.506, respectively (*P* = 1.70 × 10^− 7^).

## Discussion

Lipid metabolism gradually become a major trend in cancer research, researchers have found that lipid metabolism is reprogrammed in cancer cells, and certain lipids and their derivatives accumulate in the tumor microenvironment, which may promote cell migration and invasion. While the role of lipids in MBT remains unclear [17]. To the best of our knowledge, this is the first research found a high level of TC/HDL, ApoB and lipoprotein were associated with poor prognosis in MBT patients. And based on these 3 lipid parameters and several clinicopathological factors, we constructed a novel prognostic nomogram and a staging system which showed favorable discriminative and predictive ability. Besides, the novel staging system presented a better discriminative ability in predicting the OS than the AJCC TNM staging system in MBT cohort, which could be a great complement to the TNM staging system.

Aberrant lipid metabolism is gradually recognized as an important underlying mechanism of tumorigenesis and increasingly studies suggested that lipid and their metabolism were associated with the risk and the prognosis of cancer [9]. Since the hepatobiliary system performs a vital role in several processes of lipid transport and metabolism, it raised the question that whether lipids involved in the progression and metastasis of biliary tract cancer, and whether lipids parameters could be a new and convenient biomarker for prognosis. In our study, we revealed that TC/HDL, ApoB and lipoprotein could independently predict BMT prognosis, and these parameters could be easily measured in routine clinical laboratories and show promising diagnostic accuracy for prognosis.

Cholesterol, acts as a membrane constituent, is closely associated with membrane biosynthesis of cell proliferation. What’s more, cholesterol itself, as well as its metabolites, could regulate multiple signaling pathways of tumorigenesis, such as promoting cell proliferation by modulating hedgehog signaling proteins [18], inhibiting immune-effector cells by regulating LXR signaling [17], etc. Besides, TC/HDL was reported to be a surrogate marker of insulin resistance [19], which could further activate oncogene expression through insulin-IGF-1/IGFBP pathway [20], thus promoting tumorigenesis. Consistently, our study found that high-level TC/HDL associated with a poor prognosis. Also previous studies suggested that reduced HDL and increased cholesterol were associated with poor prognosis of cancer [10, 21]. Whether the prognostic value of the ratio of TC to HDL better than TC or HDL could be further investigated in large-scale cohorts or other cancers.

ApoB, as a ligand of LDL receptor, plays an important role in transferring lipids, participating lipoprotein metabolism and involving in tumorigenesis [22, 23], which could partially explain the association between high-level ApoB and poor MBT prognosis. Also, consistent with our results, Borgquist S, et al. reported high ApoB was associated with cancer risk in colorectal cancer, lung cancer and breast cancer in a cohort of 28,098 patients [22]. And Yan X, et al. found high-level ApoB could predict poor post-surgery prognosis in hepatocellular carcinoma [24].

While lipoprotein is a biochemical assembly of lipid and protein, which provides an efficient system for transporting lipid and regulating cellular cholesterol hemostasis. Though several subclasses of lipoprotein have been reported to be related to carcinogenesis, no study has mentioned the role of lipoprotein in cancer prognosis. Our study proved that lipoprotein could independently predict OS of MBT patients, which probably inspired more studies on the role of lipoprotein in tumorigenesis and prognosis.

What’s more, ApoB and lipoprotein were usually recognized as vital risk factor of cardiovascular diseases, and positively associated with acute myocardial infarction (AMI) and cerebral infarction (CI) [25, 26]. Thus the baseline cardiovascular disease condition and patients died from AMI and CI might introduce confounding bias and affect the accuracy of the results. Thus we also performed univariate and multivariate COX regression analysis of cardiovascular disease condition, which proved that cardiovascular disease did not interfere with the prognosis in biliary tract cancer patients. But surprisingly, we found the level of TC/HDL, ApoB and lipoprotein are not associated with cardiovascular disease in our study, which were contrast to previous studies [26, 27]. And different cut-off values of ApoB and lipoprotein among these studies might partially explain the inconsistency. Besides, our cohort is a biliary tract cancer cohort, cancer itself and multiple factors like races, could affect serum lipid levels [9].

The novel MBT prognostic nomogram first integrated serum lipid parameters in addition to biological parameters like CA199, pathological parameters like tumor differentiation and lymph node metastasis, surgical parameters like the radical cure, which suggested favorable discriminative and predictive ability. Moreover, adding lipid parameters could significantly improve the predictive accuracy and discriminative ability, which further identified by ROC curves, IDI analysis and DCA. Besides, when compared with the TNM system, this nomogram also performed better predictive performance and could distinguish patients with different risks in subgroups of various tumor types or therapeutic options. Thus, the new nomogram and staging system provide a convenient and intuitive tool to initially classify MBT patients into different prognostic stage.

There are limitations in this study. First, given it was a retrospective observational study, clinicopathological information was inevitably limited. Second, the heterogeneity of the study object is relatively large, which might attenuate the strength of evidence. However, we performed subgroup analyses to identify the performance of the novel staging system in GBC, ICC, ECC patients, which may compensate for this problem to some extent. Third, the death caused by AMI, CI or other diseases instead of cancer could introduce confounding bias which may affect the accuracy of our results, which might need detailed patients information and further study. Forth, this study only performed internal validation. To identify whether the nomogram is generalized to other institutes, external validation should be performed if possible. Fifth, this study was a single-center study, which could introduce selection bias, further large-scale multicenter prospective study is needed.

## Conclusion

A novel, serum lipid profile based nomogram for MBT patients was successfully constructed, providing new insight into the crosstalk among serum lipids and BMT prognosis. Also the nomogram could facilitate predicting prognosis and guiding individualized treatment. Besides, we established a new staging system to predict prognosis and showed favorable discriminative ability, which would be a great complement to the TNM staging system.

## Supplementary information

**Additional file 1:** **Table S1.** Demographic and clinicopathological characteristics of modified training set and validation set.

**Additional file 2:** **Figure S1.** Schoenfeld residuals versus ranked survival time for selected predictors. The X-axis represents the survival time, while the Beta values referring to TC/HDL (a), ApoB (b), lipoprotein (c), CA199 (d), tumor differentiation (e), radical cure (f), R0 (g), and lymph node metastasis (h) are shown on the Y-axis. TC, total cholesterol; HDL, high-density lipoprotein; ApoB, apolipoprotein B; CA199: carbohydrate antigen 199.

## Data Availability

Data are available from the corresponding author upon reasonable request.

## Abbreviations

ALP: Alkaline phosphatase
ALT: Alanine aminotransferase
ApoA1: ApolipoproteinA-1
APOB: Apolipoprotein B
AST: Aspartate aminotransferase
BMI: Body mass index
CA199: Carbohydrate antigen 199
CC: Cholangiocarcinoma
CI: Confidence interval
C-index: Harrell’s concordance index
DCA: Decision curve analysis
ECC: Extrahepatic cholangiocarcinoma
GBC: Gallbladder cancer
GGT: Gamma-glutamyl transpeptidase
HDL: High-density lipoprotein
ICC: Intrahepatic cholangiocarcinoma
IDI: Integrated discrimination improvement
IQR: Interquartile range
LDH: Lactate dehydrogenase
LDL: Low-density lipoprotein
MBT: Malignant biliary tumor
OS: Overall survival
PUMCH: Peking Union Medical College Hospital
R0: Histologically negative surgical margin
ROC: Time-dependent receiving operative characteristics
TBIL: Total bilirubin
TC: Total cholesterol
TG: Triglycerides
