# Supplementary material for: Integrated analysis of serum lipid profile for predicting clinical outcomes of patients with malignant biliary tumor
Source: BMC Cancer. 2020 Oct 9;20:980. doi: 10.1186/s12885-020-07496-8 (PMC7547451; doi:10.1186/s12885-020-07496-8)
Supplement: Supplementary file 1 — Additional file 1: Table S1. Demographic and clinicopathological characteristics of modified training set and validation set. [file 12885_2020_7496_MOESM1_ESM.docx]

**Table S1** Demographic and clinicopathological characteristics of modified training set and validation set.

| Characteristics | Training set, n (%) | | Validation set, n (%) |
| --- | --- | --- | --- |
| Total, N | 152 (100) | | 100 (100) |
| Tumor type |  | |  |
| GBC | 41 (27.0) | | 25 (25.0) |
| ICC | 29 (19.1) | | 18 (18.0) |
| ECC | 82 (53.9) | | 57 (57.0) |
| Age (years) |  | |  |
| ≤ 60 | 66 (43.4) | | 43 (43.0) |
| > 60 | 86 (56.6) | | 57 (57.0) |
| Sex |  | |  |
| Female | 73 (48.0) | | 50 (50.0) |
| Male | 79 (52.0) | | 50 (50.0) |
| BMI at diagnosis, kg/m^2^ |  | |  |
| < 18.5 | 8 (5.3) | | 4 (4.0) |
| 18.5-22.9 | 55 (36.2) | | 38 (38.0) |
| ≥23 | 76 (50.0) | | 49 (49.0) |
| NA | 13 (8.6) | | 9 (9.0) |
| Alcohol | |  | |
| Yes | 37 (24.3) | | 75 (75.0) |
| No | 115 (75.7) | | 25 (25.0) |
| Fatty liver |  | |  |
| Yes | 10 (6.6) | | 3 (3.0) |
| No | 142 (93.4) | | 97 (97.0) |
| Jaundice |  | |  |
| Yes | 77 (50.7) | | 44 (44.0) |
| No | 75 (49.3) | | 56 (56.0) |
| Tumor size, cm |  | |  |
| d ≤ 2  2 < d ≤ 3  3 < d ≤4  d > 4  NA | 69 (45.4)  35 (23.0)  12 (7.9)  31 (20.4)  5 (3.3) | | 43 (43.0)  24 (24.0)  15 (15.0)  13 (13.0)  5 (5.0) |
| Lymph node metastasis |  | |  |
| Yes | 46 (30.3) | | 35 (35.0) |
| No | 106 (69.7) | | 65 (65.0) |
| Extrahepatic involvement |  | |  |
| Yes | 93 (61.2) | | 65 (65.0) |
| No | 59 (38.8) | | 35 (35.0) |
| Intrahepatic involvement |  | |  |
| Yes | 52 (34.2) | | 30 (30.0) |
| No | 100 (65.8) | | 70 (70.0) |
| Gallbladder involvement |  | |  |
| Yes | 48 (31.6) | | 30 (30.0) |
| No | 104 (68.4) | | 70 (70.0) |
| AJCC 8^th^ TNM stage  I  II  III | 54 (35.5)  46 (30.3)  52 (34.2) | | 40 (40.0)  27 (27.0)  33 (33.0) |
| Tumor differentiation |  | |  |
| Undifferentiation | 2 (1.3) | | 0 (0.0) |
| Low | 14 (9.2) | | 10 (10.0) |
| Low-moderate | 45 (29.6) | | 27 (27.0) |
| Moderate | 41 (27.0) | | 29 (29.0) |
| Moderate-high | 3 (2.0) | | 6 (6.0) |
| High | 47 (30.9) | | 28 (28.0) |
| Radical cure |  | |  |
| Yes | 96 (63.2) | | 61 (61.0) |
| No | 56 (36.8) | | 39 (39.0) |
| R0 |  | |  |
| Yes | 103 (67.8) | | 65 (65.0) |
| No | 49 (32.2) | | 35 (35.0) |
| TC level, mmol/L |  | |  |
| ≤ 7.28 | 124 (81.6) | | 90 (90.0) |
| > 7.28 | 28 (18.4) | | 10 (10.0) |
| TG level, mmol/L |  | |  |
| ≤ 3.14 | 128 (84.2) | | 87 (87.0) |
| > 3.14 | 24 (15.8) | | 13 (13.0) |
| HDL level, mmol/L |  | |  |
| ≤ 0.95 | 85 (55.9) | | 51 (51.0) |
| > 0.95 | 67 (44.1) | | 49 (49.0) |
| LDL level, mmol/L |  | |  |
| ≤ 1.96 | 19 (12.5) | | 8 (8.0) |
| > 1.96 | 133 (7.5) | | 92 (92.0) |
| TC/HDL |  | |  |
| ≤ 10.08 | 112 (73.7) | | 75 (75.0) |
| > 10.08 | 40 (26.3) | | 25 (25.0) |
| TG/HDL |  | |  |
| ≤ 4.16 | 114 (75.0) | | 74 (74.0) |
| > 4.16 | 38 (25.0) | | 26 (26.0) |
| ApoA1 level, g/L |  | |  |
| ≤ 1.11 | 75 (49.3) | | 50 (50.0) |
| > 1.11 | 77 (50.7) | | 50 (50.0) |
| ApoB level, g/L |  | |  |
| ≤ 0.90 | 100 (65.8) | | 65 (65.0) |
| > 0.90 | 52 (34.2) | | 35 (35.0) |
| Lipoprotein level, g/L |  | |  |
| ≤ 72 | 85 (55.9) | | 56 (56.0) |
| > 72 | 67 (44.1) | | 44 (44.0) |
| Albumin level, g/L |  | |  |
| ≤ 35 | 21 (13.8) | | 15 (15.0) |
| > 35 | 128 (84.2) | | 85 (85.0) |
| NA | 3 (2.0) | | 0 (0.0) |
| ALT level, U/L |  | |  |
| ≤ 40 | 60 (39.5) | | 32 (32.0) |
| > 40 | 92 (60.5) | | 68 (68.0) |
| AST level, U/L |  | |  |
| ≤ 40 | 71 (46.7) | | 37 (37.0) |
| > 40 | 79 (52.0) | | 63 (63.0) |
| NA | 2 (1.3) | | 0 (0.0) |
| GGT level, U/L |  | |  |
| ≤ 45 | 35 (23.0) | | 25 (25.0) |
| > 45 | 115 (75.7) | | 75 (75.0) |
| NA | 2 (1.3) | | 0 (0.0) |
| ALP level, U/L |  | |  |
| ≤ 135 | 62 (40.8) | | 33 (33.0) |
| >135 | 88 (57.9) | | 67 (67.0) |
| NA | 2 (1.3) | | 0 (0.0) |
| LDH level, U/L |  | |  |
| ≤ 250 | 130 (85.5) | | 87 (87.0) |
| > 250 | 19 (12.5) | | 13 (13.0) |
| NA | 3 (2.0) | | 0 (0.0) |
| TBIL level, umol/L |  | |  |
| ≤ 17.1 | 61 (40.1) | | 35 (35.0) |
| > 17.1 | 91 (59.9) | | 65 (65.0) |
| CA199 level, U/mL |  | |  |
| ≤ 37 | 48 (31.6) | | 31 (31.0) |
| > 37 | 104 (68.4) | | 69 (69.0) |
| Overall survival, months | 18.9 (10.1-34.7) | | 25.0 (11.0-40.3) |

ALP, alkaline phosphatase; ALT, alanine aminotransferase; ApoA1, apolipoproteinA-1; APOB, apolipoprotein B; AST, aspartate aminotransferase; BMI, body mass index; CA199: carbohydrate antigen 199; CC, cholangiocarcinoma; ECC, extrahepatic cholangiocarcinoma; GBC, gallbladder cancer; GGT, gamma-glutamyl transpeptidase; HDL, high-density lipoprotein; ICC, intrahepatic cholangiocarcinoma; LDL low-density lipoprotein; NA, not accessible; TBIL, total bilirubin; TC, total cholesterol; TG, triglycerides.
