# Supplementary figures and images for: Integrated analysis of serum lipid profile for predicting clinical outcomes of patients with malignant biliary tumor
Source: BMC Cancer. 2020 Oct 9;20:980. doi: 10.1186/s12885-020-07496-8 (PMC7547451; doi:10.1186/s12885-020-07496-8)

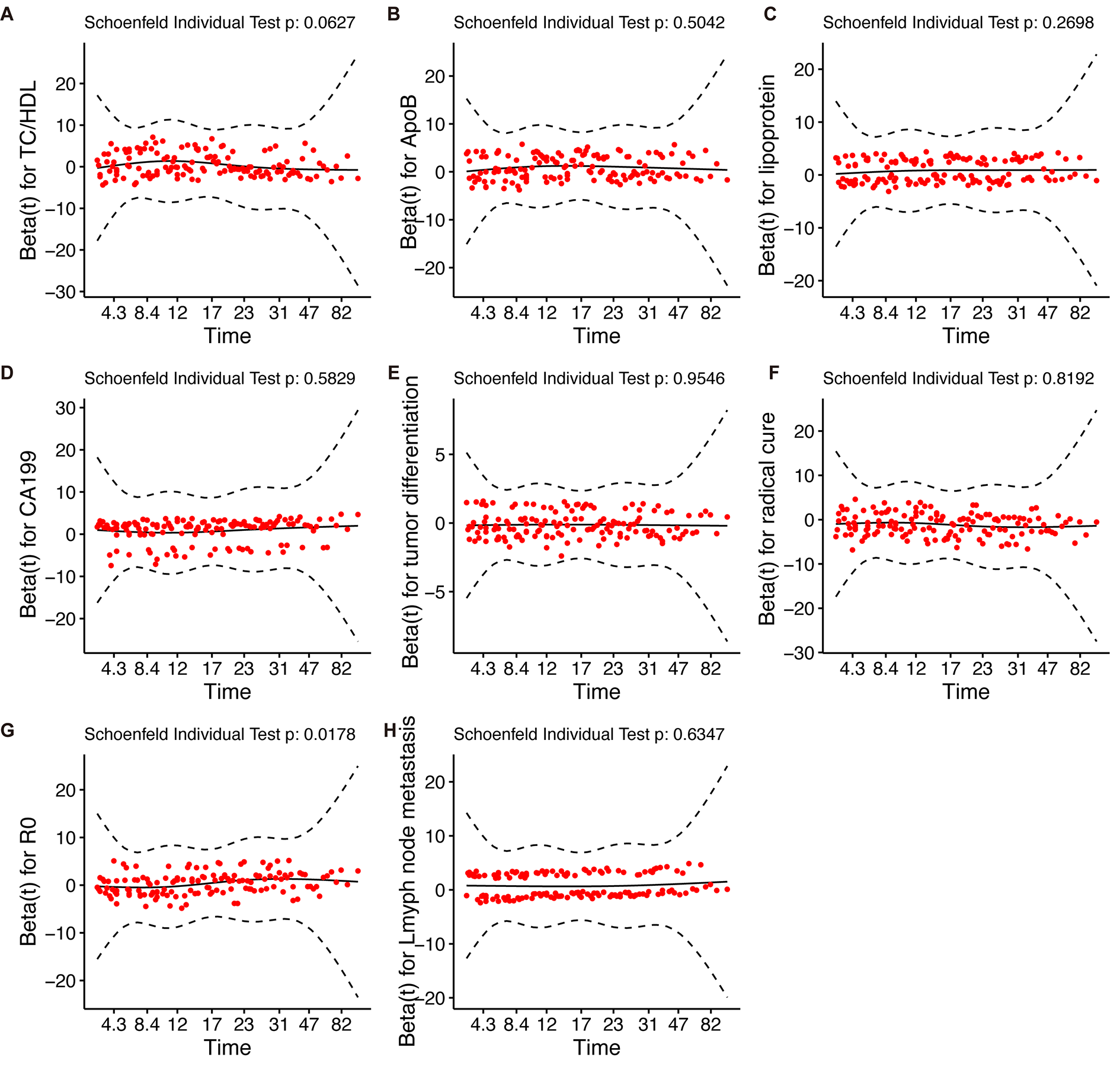

Supplement: Supplementary file 2 — Additional file 2: Figure S1. Schoenfeld residuals versus ranked survival time for selected predictors. The X-axis represents the survival time, while the Beta values referring to TC/HDL (a), ApoB (b), lipoprotein (c), CA199 (d), tumor differentiation (e), radical cure (f), R0 (g), and lymph node metastasis (h) are shown on the Y-axis. TC, total cholesterol; HDL, high-density lipoprotein; ApoB, apolipoprotein B; CA199: carbohydrate antigen 199. [file 12885_2020_7496_MOESM2_ESM.tif]
